# Human Action Recognition: A Taxonomy-Based Survey, Updates, and Opportunities

**DOI:** 10.3390/s23042182

**Published:** 2023-02-15

**Authors:** Md Golam Morshed, Tangina Sultana, Aftab Alam, Young-Koo Lee

**Affiliations:** 1 Department of Computer Science and Engineering, Kyung Hee University, Global Campus, Yongin-si 17104, Republic of Korea; 2 Department of Electronics and Communication Engineering, Hajee Mohammad Danesh Science & Technology University, Dinajpur 5200, Bangladesh; 3 Division of Information and Computing Technology, College of Science and Engineering, Hamad Bin Khalifa University, Doha P.O. Box 34110, Qatar

**Keywords:** human action recognition, computer vision, deep learning, hand-crafted, taxonomy, survey

## Abstract

Human action recognition systems use data collected from a wide range of sensors to accurately identify and interpret human actions. One of the most challenging issues for computer vision is the automatic and precise identification of human activities. A significant increase in feature learning-based representations for action recognition has emerged in recent years, due to the widespread use of deep learning-based features. This study presents an in-depth analysis of human activity recognition that investigates recent developments in computer vision. Augmented reality, human–computer interaction, cybersecurity, home monitoring, and surveillance cameras are all examples of computer vision applications that often go in conjunction with human action detection. We give a taxonomy-based, rigorous study of human activity recognition techniques, discussing the best ways to acquire human action features, derived using RGB and depth data, as well as the latest research on deep learning and hand-crafted techniques. We also explain a generic architecture to recognize human actions in the real world and its current prominent research topic. At long last, we are able to offer some study analysis concepts and proposals for academics. In-depth researchers of human action recognition will find this review an effective tool.

## 1. Introduction

**Figure 1:**
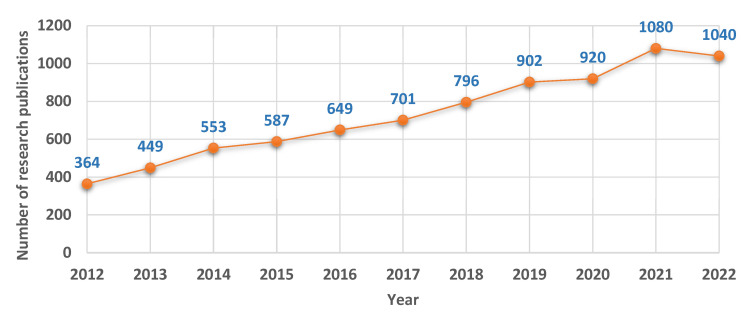
Number of research publications on human action recognition over the last ten years.

Researchers are showing increasing interest in human activity recognition, as shown by the growing number of research publications in the field over the last ten years (Figure 1). The reason behind this incremental trend is the many different areas in which it is used, including human–computer interaction (HCI), surveillance cameras, virtual reality (VR), and elder care. By looking at research papers published in computer vision and machine learning journals and conferences, we can see a trend in this direction. Even though the number of publications in this field has been going up, the exact rate of growth may vary, depending on things like how popular certain sub-topics are within human action recognition, changes in funding and resource allocation, and the development of new and innovative methods. However, anyone can find academic articles about human action recognition by searching online academic databases like Google Scholar, MDPI, IEEE Xplore, or ACM Digital Library. These databases track and index research publications in many fields, including human action recognition.

The widespread use of computer vision for human activity identification is an important step toward implementing practical solutions. For instance, in healthcare contexts, it may make it easier for technology to monitor and analyze the progress of patients who are undergoing motion rehabilitation, by removing the demand for wearing sensors. It may allow the identification of elderly in an emergency state, such as having fallen down. It may give the essential information to alert a robot that has been trained to aid in such situations or alert an appropriate organization [1]. If we take academia as an example, this technology might be utilized to expand the capabilities of robots, giving them the ability to enhance social interaction skills in autistic spectrum disorder cases [2]. Human activity recognition is particularly useful for sports areas, since it can record and evaluate the performance of players, allowing for the further growth of their abilities. The notion that robots can execute desired tasks by reading human intentions in human–robot interaction or cooperation situations is applicable. By using human activity detection, it could also be used in virtual reality and augmented reality apps to enable the user to use augmented reality in a natural way.

There has been a lot of progress made in the field of human action recognition in recent years, with a number of interesting new products appearing on the market. Here are a few examples:

- Smart cameras: These cameras use algorithms, based on artificial intelligence (AI) and machine learning (ML), to track and identify people’s actions in real time.
- Wearable Devices: Wearable technology uses sensors to monitor the wearer’s every move, allowing for accurate recognition of common physical motions like running, jumping, and walking.
- Health and Fitness Apps: Apps for health and fitness track and analyze user data using artificial intelligence and machine learning algorithms to make suggestions and give feedback, based on specific activities like running, cycling, and swimming.
- Automated Surveillance Systems: For security and safety reasons, automated surveillance systems are available that use artificial intelligence and machine learning algorithms for human action identification.
- Human–computer interaction: systems that employ human action recognition for human–computer interaction are available, with examples including gesture recognition in gaming and virtual reality.

These are just a few examples of new products made to recognize human actions. The field is dynamic, so it is reasonable to expect plenty of new and interesting advances in the near future.

Understanding human activity is more difficult than ever due to changing online environments, occlusion, different viewpoints, execution pace and biometric changes. Online Adaptation is the capacity to identify activities that occur in continuous video feeds and respond immediately by classifying the event. When it comes to action identification, the traditional method often puts an emphasis on categorizing the manually-clipped actions, and contrasts with online action recognition. While traditional action recognition is easier, in that it only requires classifying the manually-trimmed actions, online action recognition is more difficult. It needs to detect and recognize the occurrences of actions without classifying them, and it must be done in the presence of only partial actions. Occlusion may create difficulty in differentiating between various bodily parts, due to inter-occlusion and self-occlusion [3]. As the human body alters with varying sizes, appearances, shapes, and distances, viewpoint and biometric variability results in significant intra-class variance, which, in turn, impacts the performance of algorithms. It is also possible that these differences in execution rates are caused by differing performance styles and speeds.

The recent advancement of Convolutional Neural Network (CNN) has led to remarkable progress in human action recognition in videos. Several tasks. like classification, segmentation, and object detection, have significantly improved through CNN. Unfortunately, the impact of this progress is mostly on image-based tasks. There was not initially as much focus on the video domain, due to the inability of neural network models to capture temporal information in video and due to a lack of large data sets.

There have been many research articles summarizing work employing methods of human activity recognition in the computer vision field. A broad description of available RGB action datasets is given by Zhang et al. [4]. Chen et al. [5] evaluated human action recognition techniques that make use of the concept of depth. Kinect sensor-based motion detection applications were demonstrated by Lu et al. [6]. In [7,8], skeleton-based action recognition algorithms were discussed with multiple anatomical characteristics. In addition, there are other reviews on activity recognition, such as [9,10]. The paper by Zhu et al. [11] primarily evaluated RGB data-based human activity identification.

The study of recognizing and classifying human actions is referred to as “human action recognition”. Researchers in the field of recognizing human actions often look at the following types of actions:

- Daily activities, such as walking, running, jumping, sitting, standing, etc.
- Sports activities, such as basketball, soccer, tennis, etc.
- Exercise activities, such as weightlifting, yoga, aerobics, etc.
- Medical activities, such as gait analysis for patients with mobility impairments.
- Industrial activities, such as assembly line work, machine operation, etc.
- Interpersonal activities, such as handshaking, hugging, pointing, etc.
- Artistic activities, such as dancing, playing musical instruments, etc.
- Household activities, such as cooking, cleaning, etc.

**Figure 2:**
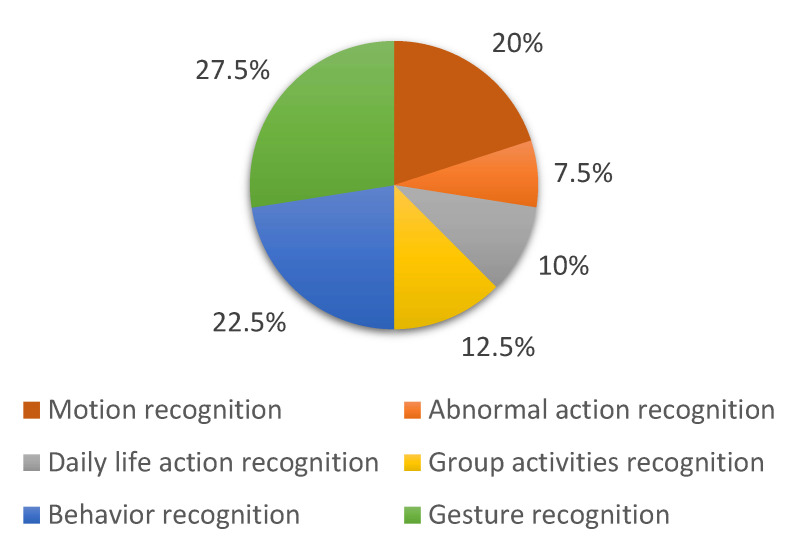
Distribution of studied types of human activities in the last ten years.

This is, by no means, a complete list, and different researchers may focus on different types of activities depending on their interests and the topic at hand. The percentage of different human activities that have been studied during the last decade is shown in Figure 2. Based on the complexities of human actions, there are four basic types of human activity [12], and they are defined as follows: actions at the atomic level, between human and object, between pairs, and within groups. The emphasis of this study is on these four kinds of action done by a single individual, or several individuals. Our study includes a thorough investigation of hand-crafted human action recognition, as well as systems based on learning. Moreover, our paper discusses practical problems and possible answers for future studies that might help improve human action recognition.

### Our Contributions

In brief, the paper’s key contributions are as follows:

- We provide a detailed introduction to human activity recognition using computer vision.
- Our comprehensive analysis of action recognition was facilitated by examining both conventional and deep learning-based approaches.
- We present a generic framework for recognizing human actions in videos.
- In order to classify all the different approaches to human action recognition, we proposed a new taxonomy, and present a detailed discussion with recent work in regard to our taxonomy.
- This study explores the challenges associated with existing approaches to actions and interactions, as well as emerging trends for possible future paths in the detection of complex human behavior and online activity.

We structured our paper as follows: Section 2 takes a look at the overall human action recognition techniques. In Section 3, we provide a generalized framework for identifying human actions. Section 4 presents research method and taxonomy for human action recognition and reviews the approaches based on feature extraction and activity types. Reviews on handcrafted methods and machine learning methods, such as deep learning, for human activity identification, as well as their capabilities in a variety of datasets, are also presented in this section. Section 5 represents the popular public datasets and approaches for human action recognition. Evaluation metrics and performances on different datasets are discussed in Section 6. Section 7 examines the issues, opportunities, and future directions of human activity recognition. Finally, Section 8 concludes and outlines potential avenues of research.

## 2. Overview

Research on human activity detection may be separated into methodologies, depending on feature extraction and the sorts of activities that are being studied. As a result of progress in machine learning studies, the human action recognition methodologies for accessible datasets may be classified as either manually-built features, using machine learning methods, or fully-automated methods, based on deep learning. It is important to remember that the fundamental objective is to acquire reliable human action characteristics, independent of the data format or processing method used. It has been suggested that spatial and temporal salient point characteristics [13,14], spatial and temporal density features [15,16] and combined trajectory features [17,18] may all be used to analyze RGB data. Human action depiction and identification using handmade features are hindered by issues like the limitations of human identification and posture estimate algorithms, camera motion, occlusion, and complex scenarios.

The use of depth sensors allows real-time, reliable human posture estimation since changes to the foreground or background have no effect on the accuracy of depth data, which enables objects to be quickly classified by their relative depth. Systems that use depth data and skeleton sequences to identify human actions have high recognition accuracy with little computational burden. In human activity recognition research, these approaches are widely used [4,19,20,21,22]. Due to the high cost and precision requirements, these techniques are useful at close range and in specialized environments. The most common types of depth cameras include triangulating (using data from more than one camera), structured light (SLT), and time of flight (TOF) cameras. Large mistakes and poor accuracy are common in outdoor conditions when using SLT or TOF depth cameras because of their sensitivity to light. While the dual camera system is cheaper, it is more difficult to use in low-light situations because of the intricacy of the depth information computation. Laser scanners, for example, may also be used to determine depth, although they are costly and unsuitable for surveillance and residential monitoring.

Microsoft’s new sensor, Azure Kinect [23], is made for applications that use artificial intelligence (AI) and the Internet of Things (IoT). The Azure Kinect sensor has a number of advanced features, such as a depth camera, RGB camera, and microphone array. The Azure Kinect sensor is part of Microsoft’s Azure AI platform and is supported by a range of machine learning models that are specifically designed for AI and IoT applications. Microsoft is empowering developers to create a wide variety of cutting-edge AI and Internet of Things applications by integrating the sophisticated capabilities of the Azure Kinect sensor with strong machine learning models.

Automated feature learning from photos, using deep learning approaches, outperforms handmade features. Numerous attempts have been made to utilize deep learning strategies to extract features using RGB, skeletal, and depth information, and this provides a fresh overview of human action detection. Data include overall outlook features, depth information, and optical flow information, and skeletal sequences may be used for multimodal feature learning [24,25,26,27] from deep networks. Human action characteristics may be learned from either single-mode or multi-modal combined data using deep learning networks. Visual patterns, as well as optical flow data, are often utilized for input into deep learning techniques, with only a small number of approaches based on skeletal and depth data being used. The field of action feature extraction has recently gained a lot of interest due to emerging high-efficiency posture estimate techniques that leverage deep learning [28,29,30], and this is currently an important study area in human activity recognition [31].

Action categorization and detection are two distinct aspects of human action recognition. Segmented videos with just a single action in them may be used to classify actions using action classification. This is done by detecting the start and finish timings of each activity, locating them in space, and classifying them as either simple or complex actions. In the early stages of human activity recognition research, the challenge of classifying actions was the primary emphasis. Human action detection research has been increasingly popular over the years as a result of the growth of associated research subjects, like human posture estimation, object recognition, and deep learning [32,33,34,35,36,37].

Recognition of human actions is the topic of extensive research. The complexity of human action may be divided into four categories: atomic level, between human and object, between pairs, and within groups [12]. Atomic action is the simplest kind of action, including just the movement of the human body’s components. Various simple motions may be used to create more complicated activities. When it comes to basic motions, “waving”, “raising a foot”, and “bending” are among the most prevalent. An individual action is defined as “walking”, “punching”, or “jumping” when it is performed by a single individual. Human-to-object interactions, like “holding a knife” or “playing piano”, are all examples of interactions. An activity that involves numerous people or things is known as a “group action” and includes parades, gatherings, meetings, fights, and other similar events. First- and second-level action recognition have been extensively studied in the past. Research into group action detection is still very much in inception, despite the increased attention it has received in the last several years.

### Significant Achievements

Numerous laboratories and companies are investing time and resources into developing systems that recognize human actions with the use of AI and ML, particularly deep learning. Lists of the most important contributions made by the many different research groups and organizations working on human action recognition are notoriously difficult to compile. Among these organizations, the following have made particularly significant contributions:

- Stanford University, USA: Convolutional Neural Networks (ConvNets) for action recognition, which have become the standard for the field [38].
- University of Oxford, UK: Developed the Two-Stream Convolutional Networks for action recognition in videos [24].
- Carnegie Mellon University, USA: Developed the Deep Structured Semantic Model for human action recognition [39].
- Max Planck Institute for Informatics, Germany: Conducted research on human action recognition in the context of egocentric videos [40].
- Ecole Centrale de Lyon, France: Through their research on deep learning-based action recognition, they have made important progress in the field. For example, they have made algorithms for action recognition that use unstructured data [41].
- National Institute of Information and Communications Technology, Japan: Conducted research on human action recognition in the context of wearable sensors [42].
- University of California, USA: Conducted extensive research on 3D human action recognition using deep learning [43].
- Chinese Academy of Sciences, China: Developed the Skeleton-based adaptive convolution models for human action recognition in videos [44].
- Technical University of Munich, Germany: Conducted research on human action recognition in the context of ego–motion representation [45].
- INRIA, France: Conducted research on human action recognition using deep learning and introduced the concept of spatiotemporal convolutional networks [46].

However, this is, by no means, an entire list, and many other groups and organizations have made important contributions to human action recognition as well.

## 3. Human Action Recognition Framework

Based on the kind of data analyzed, HAR, in earlier studies, was classified into two basic techniques: vision-based and sensor-based [47,48]. The first examines photos or videos captured by optical sensors [49,50], while the second investigates raw data from wearable sensing devices and monitoring devices [51,52]. Optical sensors may be distinguished from other kinds of sensors by the data they collect. Optical sensors, as opposed to wearable sensors, produce two-, three-, or four-dimensional pictures or videos. As a sensor-based HAR, wearable devices are an excellent example, since they are worn by the wearer to monitor and track a wide range of actions, like running or jogging, sitting, and resting [53]. A sensor, on the other hand, does not operate if a target is either too far away [54] or conducts behaviors that are not recognized by the sensor [55]. When it comes to vision-based HARs, CCTV systems have long been used [49]. Video-based systems for recognizing gestures and activities have been extensively investigated [56,57]. Furthermore, this issue is particularly advantageous to security, surveillance [58,59], and interactive applications [60,61]. Vision-based HAR has continued to be the primary focus of study in recent years, since it is more cost-effective and simpler to acquire than data captured through sensors. Therefore, this research only includes a limited, yet representational, range of research based on computer vision.

**Figure 3:**
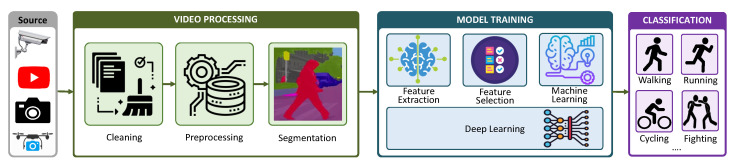
Human Action Recognition Framework.

There are four major components to the human activity recognition framework, shown in Figure 3. The first is the data collection phase, which consists of capturing data using optical sensing equipment. The second is the pre-processing phase, which includes significant pre-processing stages regarding the collected data. The third is the learning or training stage, where features are learned from the dataset using techniques like machine learning and deep learning. The fourth is the activity recognition, or classification, phase.

## 4. Research Method and Taxonomy

A complete, accurate, and up-to-date review and comprehensive taxonomy of human action recognition necessitates a methodical and rigorous approach to research. In order to conduct this survey on human action recognition, we used the following research methodology:

- Defining the scope and objectives: Goals and scope were established by first detailing what would be included in this study, which, in this case, centered on the many aspects of human action recognition. In this article, we give a brief overview of human action recognition, including where it came from, how it has changed over time, and how far it has come right to the present.
- Conducting a comprehensive literature search: We searched academic literature extensively to find studies, articles, and publications pertinent to the study of human action recognition. We used Google Scholar, MDPI, PubMed, and IEEE Xplore, among many others, to accomplish this.
- Evaluating the quality of the literature: We evaluated the quality of the literature we found by looking at aspects like the validity and reliability of the research methods used, how well the results fit with the goals of our review, and how well the data was analyzed and interpreted.
- Classifying the literature: We organized the material we collected in terms of the precise components of human action recognition we were examining, using a classification system. Methods based on feature extraction, and methods based on activity types, and so on were all included.
- Synthesizing the literature: To synthesize the literature, we summed up the main points of each research article we studied, compared and contrasted their methods and results, and added our own original thoughts and conclusions.
- Analyzing and interpreting the data: We studied and interpreted the data from the literature review in order to address the particular issue, make conclusions, and find gaps in the present body of research.

We took a methodical and exhaustive approach to authoring this review of human action recognition in order to generate a high-quality, comprehensive, and up-to-date study that offers significant insights into this interesting and rapidly evolving topic.

**Figure 4:**
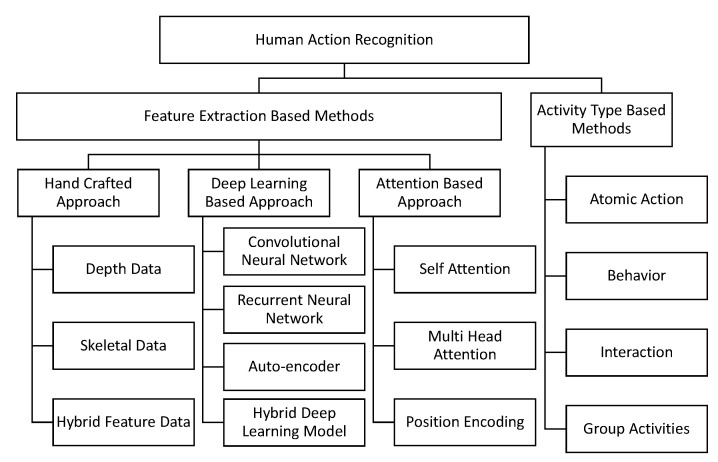
Human Action Recognition Taxonomy.

Action classification issues in the present study cover the four semantic levels of action (atomic, behavior, interaction, group). only the first two categories (atomic, behavior) of action categorization have been the subject of previous studies. There has not been a lot of work on the topic of recognizing group activity in the scientific community yet, despite the current rise in interest in interaction recognition. Action feature representation is a fundamental issue in basic categorization and in action performed by a single individual. Research on action recognition, on the other hand, focuses mostly on basic actions and single-person actions. Since this research is based on a survey, we review and examine action identifying approaches considering two perspectives: feature extraction and activity type. This is shown in Figure 4.

The next sections provide an in-depth look at human action identification techniques, including methods for extracting human action features, and methods for recognizing atomic, behavior, interaction, and group activities.

### 4.1. Feature Extraction-Based Action Recognition

#### 4.1.1. Handcrafted Representation Method

There has been a lot of recent interest in the field of handcrafted approaches to action recognition. Handcrafted feature representation has achieved great performances on different action classification problems. This approach intends to retrieve the temporal and spatial features in videos and to extract local descriptors from the frames of videos. Traditional machine learning often makes use of these features, like SVM and likelihood outline models, to recognize activities in raw videos. Handcrafted-based strategies use human perception and historical context to obtain actionable insights from data. There are typically three main stages to these kinds of strategies: (1) Segmentation of action (2) Selection of features and (3) Action classification, based on captured features. In order to build the descriptor, key features are extracted from the source video segments. The categorization is carried out via a general-purpose classifier, thereby expanding the method’s adaptability, giving rise to lower computational costs and not being dependent on large data sets for training. Depending on the data modality the hand crafted approach can be categorized in three methods: techniques based on depth, techniques based on skeletons, and techniques based on hybrid types of features.

##### Depth-Based Approaches

**Figure 5:**
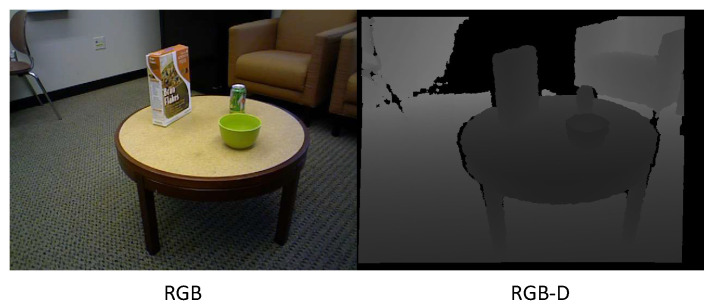
RGB and RGB-D image from RGB-D Object Dataset [68]. Pixels in an RGB-D image are used to represent how far away an item is from the screen. Those closest to the camera have the greatest pixel values, while those farthest away from the camera have the lowest.

As depth cameras and range imaging methods [62] have improved, researchers are able to more precisely execute HAR. To aid computers in recognizing human activity more precisely, RGB-D cameras gather depth information in addition to the original RGB data (as seen in Figure 5). Depth-based methods for action recognition take the depth images as input and detect the foreground to extract the human body and its corresponding movement in action. Several researchers [22,63,64,65,66] projected the depth information of an image frame in 3D, such as views from the top, front and side, so as to extract the features. In [63] 3D points from the human body were derived from depth mapping to model the corresponding postures for human action recognition. However, it is computationally costly and time consuming to process a huge amount of 3D extracted points in large datasets. A method for human action recognition was proposed by [67], in which they used depth maps to generate a Depth Motion Maps (DMM) and, then, the DMM was used to compute a Histogram of Gradients (HOG). However, Chen et al. [22] claimed that, by substituting the HOGs with sequences of DMMs, computational cost could be reduced and accuracy of action recognition improved. To deal with the diversity in action speed, a multi-temporal DMM [65] was introduced to extract motion and shape information from different range of depth sections. Bulbul et al. [64] enhanced the shape attributes of DMMs by a new feature descriptor combining Contour let Transform (CT) and HOGs. However, the surroundings of 3D points were not considered in these methods, and, thus, the necessary information for action recognition might be missed.

The 3D points collected from the surface image frame can be used to calculate the normal vectors so as to extract the motion and shape features in an action recognition model [19,69,70]. In [19] a novel descriptor for action recognition, using depth sequences, was proposed. This descriptor can capture shape and motion information simultaneously from a 4D space normal orientation histogram, using depth images, time and coordinates. A super normal vector was proposed by [69], using polynormal to encode local shape and motion information. Slama et al. [70] introduced a framework, in which they modeled local displacement features as sub-spaces lying on a Grassmann manifold to also create a density function of probability for action categorization.

On the other hand, several researchers introduced the segmentation of depth data to the point of interest and to extract the features for activity detection. Wang et al. [71] presented a method for extracting semi-local features which explored extensive sampling space to reduce noise and occlusion. A method for depicting local features surrounding the point of interest from videos was presented by [72]. In [73] a local point descriptor, obtained through sampling movement and structural features to present human activity in a depth context, was proposed. Liu et al. [74] produced spatial–temporal interest points (STIPs) using movement and structural features, extracted by means of noisy depth data. They introduced a bundle of visual words, a two-tiered model which removed noise and represented both shape and motion gestures. However, the research scope of these approaches are limited by computational cost and the need to detect interest points using all depth data from videos.

##### Skeleton-Based Approaches

**Figure 6:**
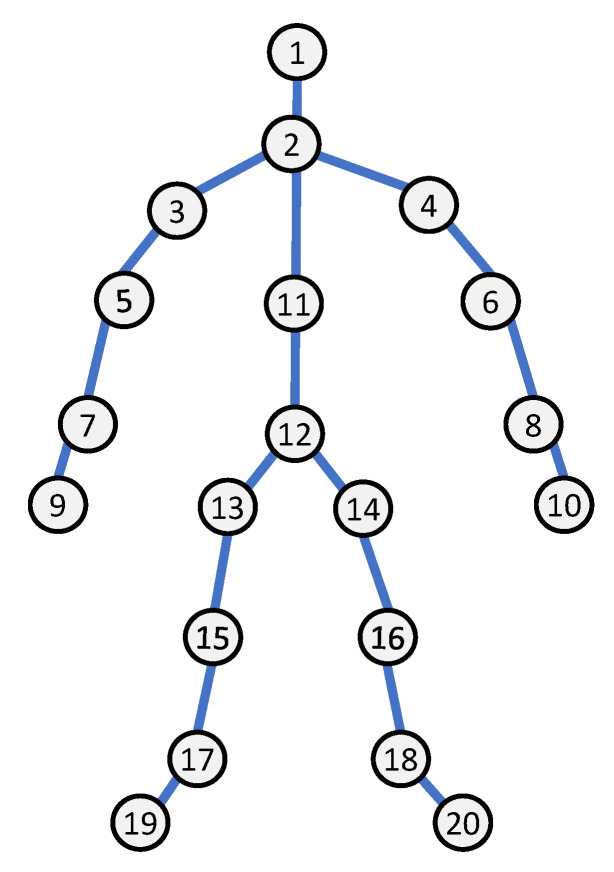
Skeleton model created with Kinect sensor in 3D. The dots indicate 20 joints, while the lines represent 19 limbs.

It is also possible to derive information on the human body’s skeleton from depth measurements, as seen in Figure 6. The low-dimensional space [75] of skeleton data makes HAR models run quicker. Exploiting depth cameras to create a 3D human joint is a promising research direction because of the wide range of potential applications. Human action representation based on body skeleton is considered an open research topic among researchers. Human body joints can be represented with 2D/3D coordinates from depth images and can be used to extract motion features by tracking their movements. Many Skeleton-based action recognition approaches are proposed by researchers and can be categorized into two types: approaches based on trajectory and approaches based on volume.

**Trajectory-Based Approach:** Approaches based on trajectory investigate the spatial and temporal movement of the human body’s skeleton to extract different features. A trajectory descriptor, based on 3D points from the human skeleton, was proposed by [76], in which they integrated several 2D points to extract the movement of all joints. In [43], human actions were represented by calculating the relative and progressive features of skeleton joint angles. To collect enough dynamic and static knowledge, Qiao et al. [77] used trajectories of local features to create a constrained time window. Devanne [78] represented action movement sequences as spots in free-form curve space by projecting the position information of skeletal joints onto a Riemannian surface. The human action classification was then accomplished by calculating the resemblance on the manifold of paths. Guo et al. [79] suggested a gradient variance-based function to reflect movement trajectories of rigid bodies in six dimensions by decomposing the human body skeletal data considering five parts. After sparsely historizing the coded skeletal representations, a support vector machine (SVM), using a chi-square kernel, was applied for action detection. To improve skeleton-based action identification, [80] proposed PoseConv3D. Instead of using a graph pattern to depict human bones, PoseConv3D uses a three-dimensional heatmap volume.

**Volume-Based Approach:** Texture, colour, pose, histograms of optical flow, histograms of directed gradients, and other features can be used in volume-based methods to reflect video as a spatial–temporal volume. Similarities between the two volumes are used to identify behavior. When the scene is noisy, volume-based techniques are ineffective. They are generally good for detecting very basic motion or gestures. Two-dimensional focus point detectors [81], include the use of techniques, such as scale invariant feature transformation (SIFT), as well as other techniques, such as corners and the Gaussian Laplacian, and were used to detect 3D interest points presented in [82]. Chaaraoui et al. [83] used an evolutionary technique to choose an optimum collection of skeletal data to create primary pose orders for every movement using dynamic time warping (DTW). Movements of five different bodily components were used by [84] to project their relative 3D structural relationship, for which human action was represented as curves. Even though there were overlapped areas of body parts, this approach could expose the concurrence of body parts, while isolating body parts could be difficult. Recently, ref. [85] specifically examined skeletal data to jointly acquire various pair-wise relational connections among various human objects, enabling group action detection.

##### Hybrid Feature-Based Approaches

A combination of multi-sensory information, including color and depth maps, as well as skeletal data, can improve detection performance. There are a number of suggested approaches that use a combination of joint and depth image features to extract the matching depth data surrounding skeletal points. A new function, called the “local occupancy pattern” (LOP), was developed by [86,87] to obtain details of the visuals of every point by means of capturing local depth data. Through embedding skeleton data into depth sequences, ref. [75] partitioned the human body into many motion sections. A discriminatory descriptor was created by combining local features derived from these motion pieces. In [88], an hierarchical hidden Markov model’s base layer was also used to correlate depth knowledge of objects around skeleton joints. Using a random forest-based fusion technique, ref. [89] coupled motion based on spatial information and interest points. In order to choose informative skeleton frames, Yang et al. [21] suggested using the cumulative movement energy feature extracted from depth maps and the minimize computational expenses by excluding noisy frames. After computing eigenjoints, they employed the non-parametric Naive Bayes Nearest Neighbor method to differentiate between various behaviors.

Some researchers suggest using RGB data in addition to a mixture of skeleton joints with depth frames. Ref. [90] modeled motion characteristics using skeleton joints to provide descriptions of appearance signals. A linked, hidden, conditionally randomized field model [91] was presented for learning the dormant relationship of ocular characteristics through depth and RGB sources. The temporal sense within each modality was maintained in this model when learning the association between two modalities. For action recognition, ref. [92,93] produced spaces by projecting details using RGB and depth photos and then using layers to keep individuals’ places discreet, indicating that information and similarity from multiple sources may be exchanged to minimize noise and increase efficiency. State-of-the-art methods for HAR, based on handcrafted features, are shown in Table 1.

#### 4.1.2. Deep Learning Representation Method

Deep learning is a branch of machine learning that uses hierarchical algorithms to learn high-level abstractions from data. It is a prominent approach that has been extensively used in conventional AI domains, including semantic parsing, transfer learning, natural language processing, computer vision, and many others. The appearance of large, high-quality, and publicly accessible marked datasets, as well as the empowerment of parallel GPU computation, allowing transformation from CPU-based to GPU-based training, and, thereby, facilitating substantial acceleration in deep model training, are two of the most prominent factors that contributed to deep learning’s massive boost. Neural networks, hierarchical probabilistic structures, and a number of unsupervised and supervised feature learning algorithms are all part of the deep learning family of techniques. Deep learning approaches have recently sparked interest due to their ability to get better performance than state-of-the-art strategies in a variety of activities, as well as the availability of diverse data from various sources.

Computer vision has accomplished noteworthy outcomes by shifting from hand crafted features to deep learning-based features. Deep Learning-based action recognition is gaining much attention these days because of its remarkable performance and power in extracting features from multi-dimensional datasets. Unlike the classical machine learning hand crafted method, where features need to be modeled to recognize human action, deep learning methods insert each feature into a deep network as input and learn the complex information through several layers. Deep learning models are very much data hungry and computationally costly in the training phase. The objective of these models is to learn various types of representation that offer an automated extraction of necessary features for action recognition. Action recognition approaches using deep learning structure could be classified as Convolutional Neural Networks, Autoencoders, Recurrent Neural Networks, and Hybrid Models.

##### Convolutional Neural Networks (CNN)s

**Figure 7:**
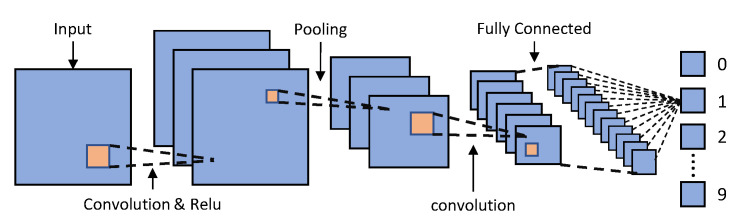
General CNN architecture.

Convolutional Neural Networks (CNNs) are widely recognized as a leading technique in the field of deep learning, in which several layers are robustly equipped. It has been shown to be highly accurate and is the most extensively employed in many computer vision tasks. Figure 7 depicts the general CNN architecture’s flow. There are three primary types of neural network layers that make up a CNN, and they are, respectively, referred to as convolutional, pooling, and fully connected. Different layers have different functions. A forward process and a backward process are used to train the network. The forward process’s primary objective is to represent the input image in each layer using the current parameters (weights and bias). The losses are then calculated using predicted and actual values.

The majority of CNN-based action recognition approaches include converting the locations or translation of skeletal components into visual representations, which are then classified using CNN. A linear interpolation method was used in [110] to build three-dimensional skeleton joints to provide four sets of 2D maps, each representing a different joint’s location in space, using a linear interpolation function. To make use of the generated range mapping in conjunction with AlexNet, the action was categorized. Ke et al. [111] used the relative locations to make three clips of grayscale images. The local structure knowledge was integrated to detect action, by loading grayscale images into the VGGNet that had already been trained, thereby producing a network capable of learning many tasks at once. Despite the fact that an overall image scaling procedure might introduce additional noise for the network, ref. [112] suggested simply inputting a skeletal image to a revised CNN-based Inception–ResNet structure for activity identification. The disadvantage of this approach is that it assumes that each operation has a set number of input frames. In [27,113] they encoded the spatial and temporal details of three dimensional skeletal orders across three combined trajectories, depending on the three perspectives (top view, front view, and side view). Three ConvNets, trained using trajectory maps, were fused late in the process to get the classifications. Xie et al. [114] Morshed used a temporal readjustment inside a residual training module to reduce the differences across skeletal sequences in the spatial–temporal domain and then modeled this data using convolutional neural networks in action detection.

Unlike traditional approaches, Yan et al. [31] built a deep graph neural network that automatically extracted the spatiotemporal pattern from the skeletal data by using joint coordinates and estimated confidences as graph nodes. Using a neural network design, Huang et al. [115] demonstrated that a non-Euclidean Lie cluster configuration [116] might be included in deep learning by extracting temporally coupled Lie group structures for action identification. Liu et al. [117] presented a method wherein a body structure transformation picture with figure posture was created to decode snippets of motion. To mitigate domain-shift and enhance the model’s generalizability, Tang et al. [118] developed a self-supervised training framework in an unsupervised space adaptation environment that split and permuted individual time sequences or parts of the body. Ref. [119] presented a technique, termed “nonuniform temporal aggregation” (NUTA), that combines data from only informative time intervals, allowing both localized and clip-level data to be merged. Ref. [120] created a secondary, lightweight network on top of the main one and had them make predictions about each other’s pseudo-labels. In order to acquire underlying long-term temporal reliance in an adaptable fashion, ref. [121] suggested a unique Temporal Relocation Module (TRM). In order to guarantee full and comprehensive activity detection by oversampling, ref. [122] offered the notion of overlapped spatiotemporal cubes, which provided the backbone of activity suggestions. The current state-of-the-art CNN-based approaches are summarized in Table 2.

##### Recurrent Neural Networks (RNNs)

**Figure 8:**
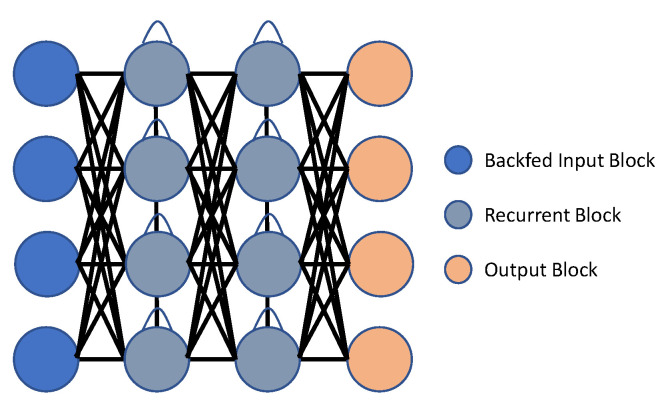
RNN block diagram.

**Figure 9:**
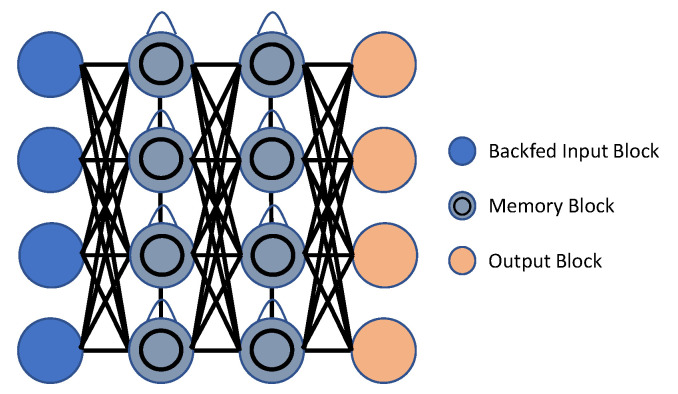
LSTM block diagram.

In comparison to CNNs, Recurrent Neural Networks (RNNs) are able to accurately model temporal data. Current approaches based on RNN often use LSTM to handle lengthy action sequences, because this architecture may circumvent the overall disappearing of gradient issue through using a gathering function to retrieve the effective cache size for a load pattern. Figure 8 and Figure 9 show the basic block diagram of RNN and LSTM, respectively. RNN-based approaches, rather than transferring motion information to images, refer to joints, or the relationship between joints, as a data source.

Differential RNNs with gating added to the LSTM were proposed by Veeriah et al. [138] to represent the variations of salient movements. A wide range of characteristics compiled from many frames were input into the proposed LSTM framework. An end-to-end hierarchical recurrent neural network (RNN) that combined features from five human limbs was proposed for behavior detection by Du et al. [139,140]. However, as pointed out in [141], this procedure neglected to take into account the connection between non-adjacent parts. Shahroudy et al. [142] built a part-aware LSTM using the human body structure. By linking together different types of part-based memory cells, the 3D skeletal series was used to teach the relationships between non-adjacent parts. Action recognition in RGB video was accomplished by Mahasseni et al. [143] by layering a regularized long short-term memory (LSTM) network over a deep CNN. They proposed utilizing the 3D skeletal sequence from several acts to regularize the network, reasoning that thr additional data would make up for any gaps in the video’s coverage. Zhu et al. [144] input a skeletal point into the multilayer LSTM model generalization for developing co-occurring properties during behavior identification. To analyze the many geometric relational properties of all joints, and to determine behavior, Zhang et al. [141] employed a stacked three-layer LSTM. After noticing the lack of precision while transitioning 3D skeletal joints into the individual position method, Zhang et al. [145] presented the viewing-adaptable RNN–LSTM structure as a means of dealing with viewpoint disparities. By using the global LSTM memory unit, Liu et al. [146] created a whole–specific condition sensible LSTM that intelligently focused on informative joints across frames. The attentional potential was further enhanced by using a repeating attention mechanism that enhanced identification accuracy by decreasing the overall noise of unrelated joints.

In contrast to prior RNN-based models, that only described the time domain of a skeleton, Liu et al. [26] presented a hierarchical layout-focused traversing strategy to manage the spatially adjacent map depicting the skeletal joint. Furthermore, a confidence gate was presented to filter out noise and occlusion in three-dimensional skeletal features. For behavior detection, Song et al. [147] recommended integrating joint-selection gates into the spatially focused structure and frame-selection gates into the temporal framework. Both the spatial embodiment of skeletons and their temporal dynamics were modeled using the two-stream RNN design presented by Wang et al. [148]. The extra spatial RNN modeled mutual spatial dependence by taking motion information into account. Si et al. [149] used a residue mapping-based connection for labeling individual body parts as nodes, thereby capturing the structural interaction between components at each frame. Next, a temporally stacked learning system, comprised of a 3 layer LSTM, was used to represent the time-series development of the joints.

##### Autoencoders

**Figure 10:**
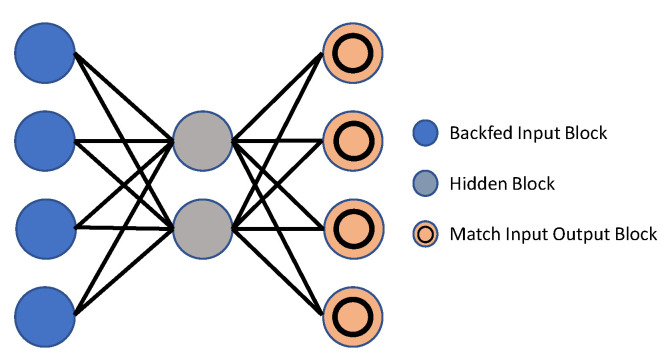
Autoencoder block diagram.

An autoencoder [150] is a type of neural network that is used to learn effective encodings. An autoencoder is programmed to recreate its own inputs, rather than training the network to predict any target value. As a result, the outcome vectors have the same dimensions as the input vectors. The autoencoder is improved by minimizing the replication error during the operation, and the learned function is the corresponding code. In most cases, a single layer is incapable of extracting the discriminative and representative characteristics of raw data. To achieve their goal, researchers now use a deep autoencoder, which passes the code learned in the previous autoencoder towards the next. Figure 10 shows the basic block diagram of an autoencoder. Hinton et al. [151] suggested the Deep Autoencoder (DAE), which has been extensively analyzed in recent papers [152,153,154]. A deep autoencoder is most often trained using a back-propagation variant, such as the conjugate gradient form. While this model is always quite accurate, it can become quite ineffective if errors are found in the first few layers. In light of this, the network learns to recreate the training data’s average. Pre-training the network with initial weights that estimate the final solution is meant to address this issue efficiently [151]. There are also autoencoder variants, proposed to keep the expression as “constant” as possible when the input changes. Vincent introduced a denoising autoencoder model to boost the model’s robustness [155,156], which retrieves the right input from a distorted version, requiring the model to obtain the structure of the source distribution.

With several hidden layers, a deep autoencoder is an efficient unsupervised feature representation method. The neural notion of data learning is motivated by the fact that hidden layer parameters are not manually built [157], but rather learned automatically, based on the given data. This idea inspired researchers to use DAE to learn time axis features of video sequences. During transformation, the high-dimensional deep features are squeezed down to low dimensions with minimal error. Baccouche et al. [158] suggested an autoencoder method that automatically learnt sparse over-finished spatiotemporal characteristics.

Hinton et al. [159] suggested the Restricted Boltzmann Machine (RBM), in 1986, as a generative stochastic neural network. An RBM is a Boltzmann Machine version with the requirement that the exposed and hidden units form a bipartite graph. This constraint makes training algorithms more effective, especially the gradient-based contrastive divergence algorithm [160]. Hinton [161] offered a thorough clarification, as well as a realistic method, for training RBMs. Further analysis in [162] addressed the key challenges of training RBMs, and their underlying causes, and suggested a new algorithm to overcome the challenges, which comprised of an adaptive learning ratio and an improved gradient. The model estimated binary units with noise rectified linear units to conserve information about comparative intensities as information passed across multiple layers of feature detectors, as described in [163]. Not only did the refinement perform well in this structure, but it was also commonly employed in numerous CNN-based architectures [164,165]. Deep Belief Networks (DBNs), Deep Energy Models (DEMs) and Deep Boltzmann Machines (DBMs) can all be built using RBMs as learning modules.

**Figure 11:**
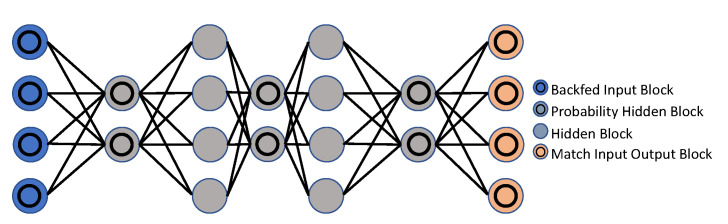
DBN block diagram.

**Figure 12:**
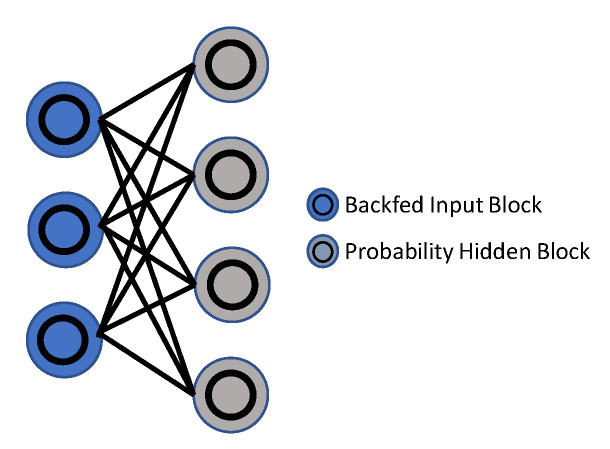
RBM block diagram.

The Restricted Boltzmann Machine (RBM) [166] is a probabilistic model, with visible and hidden variables, that uses energy as a basis for its predictions. There are visible and hidden layers, so this may be seen as an undirected, fully-connected graph. As a result of considering two successive layers as RBMs, the Deep Belief Network (DBN) is referred to when RBMs are stacked. Figure 11 and Figure 12 demonstrate the DBN and RBM architectures. Chen et al. [167] used a deep belief network (DBN) [166] model to learn symmetric spatiotemporal characteristics from videos.

##### Hybrid Deep Learning Models

**Figure 13:**
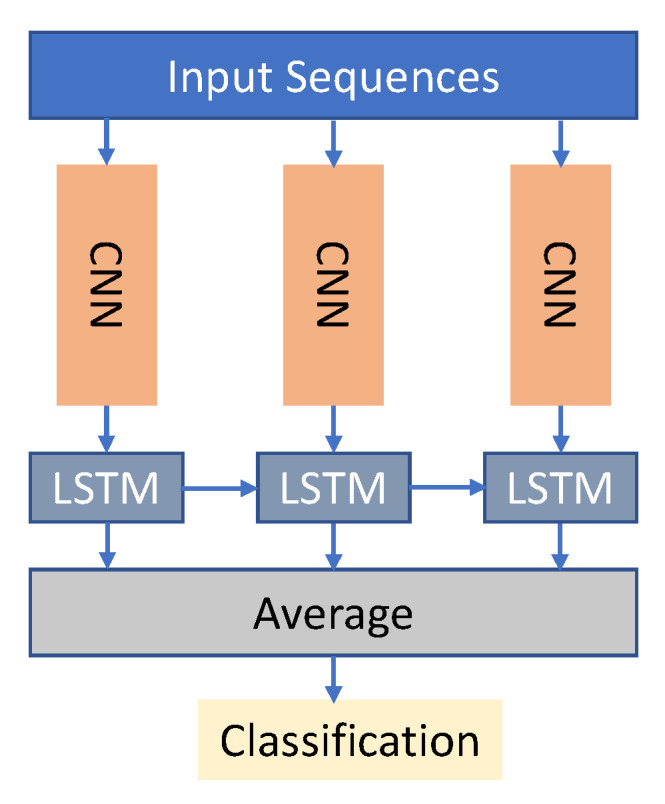
Hybrid deep learning model.

Hybrid deep learning models refer to combining two or more types of models as a means of boosting efficiency. Figure 13 depicts a sample hybrid CNN–LSTM deep learning model. For action recognition, some researchers suggested learning multi-modal features from separate networks. Three-dimensional convolutional neural networks (3DCNNs) [25,127] and bidirectional long short-term memory networks (LSTMs) were proposed by Zhang et al. [168] for acquiring spatiotemporal knowledge from multi-modal input. With the joint multi-modal characteristics in hand, the linear SVM model was used for final motion identification. To train 3 distinct CNNs for activity detection, Kamel et al. [169] proposed dividing the sequential depth information and skeletal locations into two frames. For the purpose of activity recognition, ref. [170] created a hybrid technique by combining CNN and LSTM, the former of which was used for extracting spatial characteristics and the latter for retrieving temporal features.

For the purpose of recognizing gestures across several platforms, Wu et al. [171] created the Deeply Dynamic Neural Network (DDNN). The DDNN consists of a 3DCNN that extracts spatiotemporal features in depth and RGB pictures. To avoid combining the impacts of several convolutional networks, Wang et al. [172] presented an image stream to activity mapping to join characteristics of both depth and RGB streams as the feed to ConvNets. By Analyzing Joints in the Skeleton and Depth Sequences, ref. [173] examined a privileged knowledge-based RNN system for behavior detection. Liu et al. [174] suggested learning greater attributes from raw depth images and limited features from skeleton points, such as location and angle details. For action recognition, the two kinds of features were combined and fed into SVM. Current strategies for human action identification utilizing hybrid models are outlined in Table 3.

#### 4.1.3. Attention-Based Methods

Attention models have emerged in recent years and have demonstrated promising results in a variety of difficult temporal inference tasks, including video caption recognition. After a certain job is done, it serves to increase interpretability by providing differentiable mapping from all of the output locations to the next input [188]. For the most part, a human action is comprised of several stages, such as preparation, climax, and completion. Feature learning includes unique sets of sequence frames to demonstrate various concepts in each step. As a consequence of this, while viewing a picture, one pays attention to various aspects of different areas. On the other hand, because the typical parameter learning treats each cue in the picture as equally important, it affects image recognition in a way that causes inconsistent task-specific attention recognition. Many applications use projected saliency maps to boost the degree of relevant cue correlation, thereby resulting in better recognition performance. Increasing focus is being drawn to the utilization of an attention mechanism that implicitly pays attention to related signals.

According to the study by Xu et al. [189], an attention-based architecture that learns to represent images, as well as high-quality results, in three benchmark datasets, was implemented in the image captioning application. To begin with, Bahdanau et al. [190] implemented the attention technique in computer translation and showed that performance on the problem of English-to-French translation met or exceeded the standards of the state-of-the-art phrase-based system. Some studies used RGB video to teach computer vision to recognize human actions. For example, Shikhar developed a machine learning algorithm that focused on portions of the frames and classified human actions after a few glances [182]. To further measure the uncertainty of the forecast, ref. [191] went beyond the deterministic transformer and created a probabilistic one by capturing the distribution of attention values.

The temporal attention model proposed by Z. Liu et al. studied human activities and could identify just the important frames [192]. Some studies on spatial–temporal attention examined the spatial–temporal attention design and suggested two different models: one to investigate spatial and temporal distinctiveness, and the other to explore the time complexity of feature learning [193]. These attention models were specifically designed to do action analysis in the image frames, and then mine relevant frames to find an action-related representation and combine the representation of such action-important frames to construct a powerful feature vector. Transformer is the most recent model using an attention mechanism that attracts researchers nowadays. Ref. [194] introduced GateHUB, which includes a unique position-guided gating cross-attention technique to emphasize, or downplay, portions of the past, depending on their usefulness for predicting the current frame.

**Figure 14:**
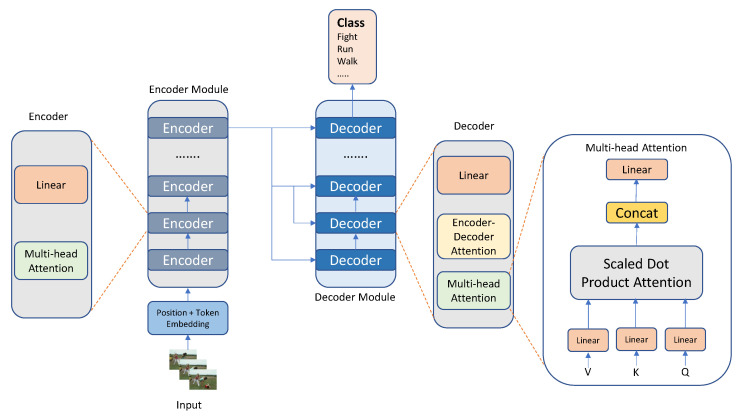
An overview of the video transformer.

**Transformer:** Natural Language Processing researchers originally developed the Transformer [195] and then showed its effectiveness in a variety of tasks [196,197,198]. Since then, it has been used in a variety of domains, from language [199,200] to vision [201]. The standard transformer is made up of many encoder and decoder components, as shown in Figure 14. Each encoder block has a self-attention layer, as well as a linear layer, and each decoder block incorporates an encoder–decoder attention layer, in addition to the other two. In recent research [202], the Point SpatioTemporal Transformer (PST2) was developed for transforming point cloud patterns. Action identification into three-dimensional point clouds may benefit from the adoption of Spatial Temporal Self Attention (STSA), which can record the spatial–temporal semantics. Ref. [203] developed various scalable model versions that factorize self-attention over the space, period, and modalities to deal with the high amount of spatiotemporal units collected from different modalities. Ref. [204] suggested processing videos using the online approach and caching “memory” for each iteration, rather than attempting to analyze more frames simultaneously, as is the case with most current systems. Ref. [205] proposed a paradigm in which many encoders would be used to represent the various angles of the video frame, using lateral connections between the encoders to fuse the data from the various angles. A transformer for video learning can be built by three approaches: self-attention, multi-head attention, and position encoding.

- Self-Attention: Self-attention is a fundamental transformer mechanism for both encoding and decoding blocks. For each video clip in the vision region, the self-attention layer takes a sequence of X (either a video clip or an entity token) and linearly converts the input into three distinct vectors: K (key), Q (query), or V (value).
- Multi-Head Attention: A multi-head attention method [195] was presented to describe the complicated interactions of token entities from diverse perspectives.
- Position Encoding: A limitation of self-attention is its inability to collect the sequence’s order information, as is the case with CNNs [206] and RNNs [207]. Position encoding [195] can be used in the encoder and decoder blocks to overcome this issue.

VideoBERT [208] was the first to use a transformer-based pre-training technique to study video-language representation. It adopted a single stream architecture, adapting the BERT [199] architecture to the multi-modal domain. Video signals and linguistic words were combined and fed into multi-layer transformers, where the model was trained on the connection between text and video. VLM (Video–Language Model) [209] is a job-agnostic model with a BERT-like inter method transformer that can take text, video, or both as input. VLM provides two new masked task schemes: Masked Token Modeling (MTM) and Masked Modality Modeling (MMM). The VATT (Video–Audio–Text Transformer) structure, proposed by [210], is an end-to-end model for learning multi-modal abstractions using direct audio, video, and text. Specific to the video’s temporal, height, and width dimensions, they divide the raw video into a series of [T/t] X [H/h] X [W/w] patches. For video–language learning, CBT. Ref. [211] suggested noise contrastive estimation (NCE) [212] as the loss goal, which maintains fine-grained video information comparable to vector quantization (VQ) and nonlinear activation loss in VideoBERT. ActBERT [213] used visual inputs, like global activity and regional objects at the local level, to help models learn video–text representations in conjunction. The Tangled Transformer block enhanced communication between diverse sources in a multi-stream paradigm. Univl [214] was the first to pre-train a model on both comprehension and generating proxy tasks. It used a multi-stream structure, with two individual transformer encoders, to incorporate video with text, an inter-modal transformer to interact completely with video and text embeddings, and a decoder for derivation processes. In the latest work, instead of computing self-attention generally, it even used spatial–temporal factorization, ref. [215] suggested a bias toward localization in visual transforms, which improved performance, while maintaining accuracy.

### 4.2. Activity Type-Based Human Action Recognition

**Figure 15:**
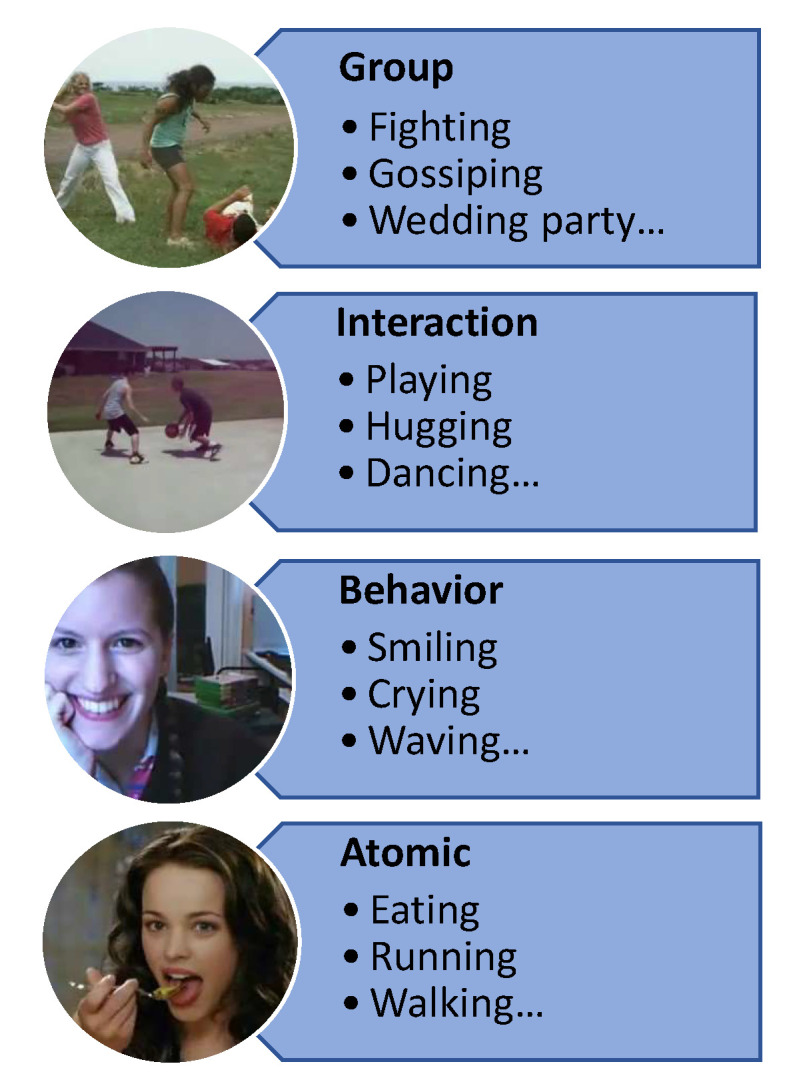
Activity type hierarchy. Images are from HOLLYWOOD 2 [216], UCF50 [217], MCAD [218], and NTU RGB+D [142] dataset.

Human activity is seen as a method of communicating, of interacting with machines, and of being engaged with the world. For this survey, we defined an activity as a specific body part or movement that is made up of numerous basic actions. These elementary actions are done sequentially throughout time. They may be done on one’s own or with others. We include a scheme in this section, ranking tasks from basic to complicated, with varying levels of complexity. This hierarchy is shown in Figure 15.

#### 4.2.1. Atomic Action

Atomic actions are simple motions at atomic levels, like lifting the hand or walking. They form the foundation for more sophisticated voluntary and purposeful movements. They are very identifiable, and have been discussed in many studies, such as [84,219,220,221]. Hand movementsm such as gesturing, may be used to convey a variety of complex thoughts or directives. “Applauding” is an example of a gesture that may be done with intent. In contrast, “covering up the face using hands when becoming uncomfortable” and “drawing off the hand upon contacting a hot substance” are unintentional. A few gestures are global, but many are connected to personal circumstances and locations. We may cite [219,222,223,224] in this field.

#### 4.2.2. Behavior

These are physical movements and actions that people exhibit in response to particular emotional and psychological circumstances and which are perceivable to others on the outside. Examples of efforts to detect such human activities include proposals found in [219,225,226].

#### 4.2.3. Interaction

These are the many forms of reciprocity in which changes occur to those participating in the contact, whether it be people or things. Human interactions, like “kissing”, and human–object interactions, like “cooking”, comprise the complex activities done as a whole. Recognizing interactions is a theme in papers like [222,227].

#### 4.2.4. Group Activities

The activities performed by a number of individuals, such as “cuddling”, are referred to as “group activities”. These actions may be more or less complicated, and they can be difficult to monitor or identify at times. Refs. [219,222] provide methods for recognizing complex activities, which make it feasible to identify complex activities. Human activities, such as weddings and parties, take place in a certain context, as do high-level activities that reflect human interactions [217,228].

## 5. Popular Datasets and Approaches

Researchers may assess their performance and verify their ideas by using publicly available datasets. Ref. [229] asserts that data files which are characterized by actions may be sorted into several groups. This includes the following: data records on movie clips, social networks, people’s ways of behaving, human postures, atomic activities, or everyday activities of daily life. Ref. [10] listed 13 data sets that might be utilized for training and testing, gathered with Kinect. We utilized popular datasets mentioned in scholarly articles and classified them by activity type: atomic action, behavior, interaction, and group activities.

### 5.1. Atomic Action Datasets

#### 5.1.1. KTH Dataset

**Figure 16:**
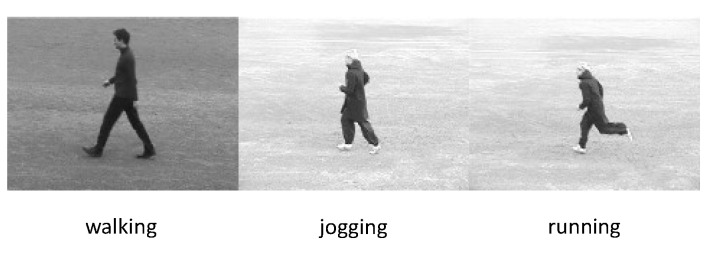
Samples from KTH dataset [230].

Sweden’s Royal Institute of Technology [230] developed the KTH dataset in 2004. The dataset has 2391 actions in four situations. It comprises 25 unique sets comprising six types of human activity (running, jogging, walking, hand clapping, boxing, and waving), repeated up to five times, by 25 participants. The average duration of the videos is 4 s long, with the videos having 4-s long segments which are shot on a static backdrop with a single camera (Figure 16).

#### 5.1.2. NTU RGB+D

**Figure 17:**
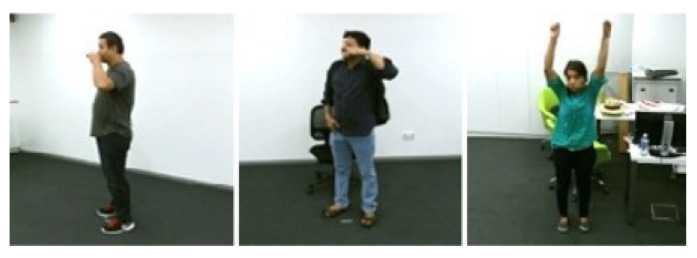
Samples from NTU RGB+D dataset [142].

This action recognition dataset was developed by Nanyang Technological University in 2016 [142]. This extensive HAR video library contains over 50,000 video clips and 4 million frames. There are 60 separate acts in it, each carried out by 40 different people, encompassing health-related and social activities. The dataset was captured using 3 Microsoft Kinect v2 devices at the same time. Its uniqueness is explained by the large number of viewing angles (80) from which it was collected. You may get an expanded variant of this dataset now [231]. In the extension, there are 120 different acts made by 106 different people (Figure 17).

#### 5.1.3. MSR Action 3D

**Figure 18:**
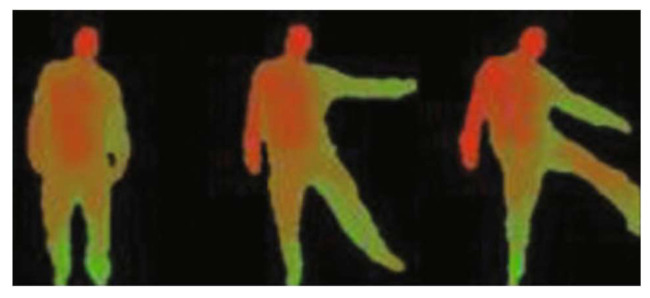
Samples from MSR Action 3D dataset [63].

The MSR Action 3D was developed at Microsoft Research Redmond by Wanqing Li [63]. It holds a total of 567 sequences of depth maps of 10 people going through 20 different types of actions twice or three times. To record the sequences, a Kinect device was used (Figure 18).

### 5.2. Behavior Dataset

#### Multi-Camera Action Dataset (MCAD)

**Figure 19:**
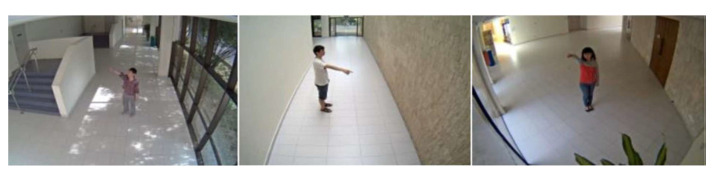
Samples from MCAD dataset [218].

NUS-National University of Singapore created this dataset in 2016 [218]. Specifically, it was created to assess the open-view categorization issue in a monitoring context. A total of 20 individuals participated in the recording of 18 daily activities that were derived out of KTH, TRECIVD, and IXMAS datasets utilizing five cameras. Every activity was performed by an individual eight instances for each camera (four times throughout the day and four times in the nighttime) in order to capture it (Figure 19).

### 5.3. Interaction Dataset

#### 5.3.1. MSR Daily Activity 3D Dataset

**Figure 20:**
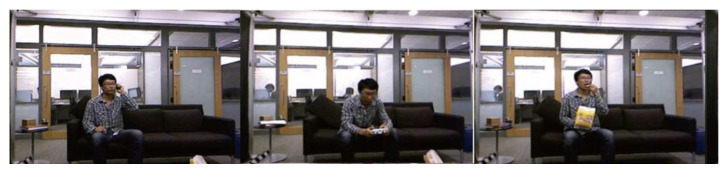
Samples from MSR Daily Activity 3D dataset [87].

Jiang Wang, a researcher at Microsoft Research in Redmond, developed this [87]. The footage comprises 320 depth maps, joint locations for each skeletal joint, and RGB clips of 10 people (both men and women) doing various tasks, such as eating, drinking, reading, and doing household chores (Figure 20). There are two stages to every activity: standing and sitting.

#### 5.3.2. Multi-Camera Human Action Video Dataset (MuHAVI)

**Figure 21:**
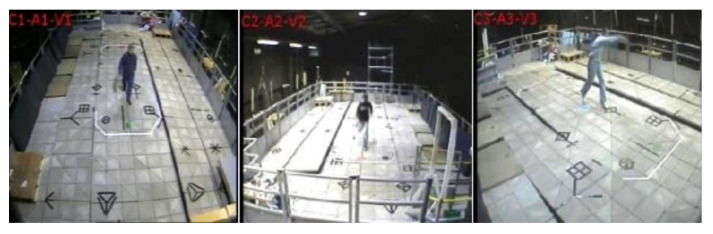
Samples from MuHAVI dataset [232].

Kingston University developed this in 2010 [232]. It focuses on human activity recognition techniques that use silhouettes. The videos used are of 14 performers doing their respective action scenes 14 times. This was done by using eight non-synchronized cameras placed on the platform’s four sides and four corners (Figure 21).

#### 5.3.3. UCF50

**Figure 22:**
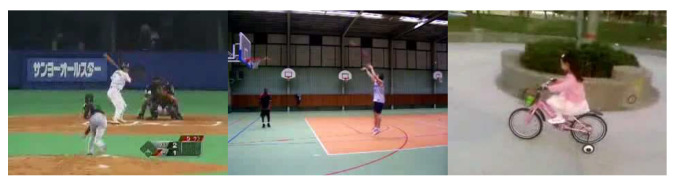
Samples from UCF50 dataset [217].

The UCF50 was developed by the University of Central Florida’s computer vision research institute in 2012 [217]. The theme for this project is that it is made up of 50 action classes, all taken from genuine YouTube videos. As an extension of the 11-category YouTube activity dataset (UCF11), this dataset features a wider variety of action-oriented videos (Figure 22).

### 5.4. Group Activities

#### 5.4.1. ActivityNet Dataset

**Figure 23:**
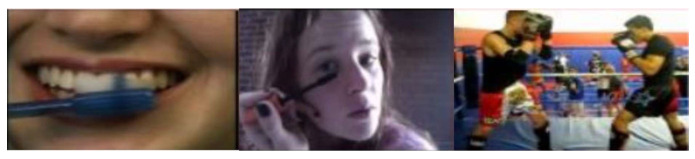
Samples from ActivityNet dataset [233].

This was introduced in 2015 [233]. The whole 849-h collection of films is used to demonstrate over 200 different activities, and each activity class has 137 unfiltered videos. There are three types of algorithms for categorizing human activity: unmodified video classification, activity classification with no filtering, and activity detection. As complicated human activities, the dataset includes many varied scenarios and movements (Figure 23).

#### 5.4.2. The Kinetics Human Action Video Dataset

**Figure 24:**
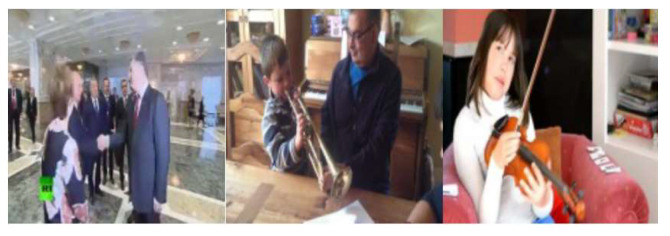
Samples from kinetics dataset [234].

The DeepMind team developed this in 2017 [234]. There were 400 human activity categories in the first version (Kinetics 400) and each one had a minimum of four hundred YouTube video snippets featuring a wide range of activities. An improved version from the earlier Kinetics 400 collection, called Kinetics 600 dataset, intended to capture around 600 human action classes. Each action class includes a minimum of 600 video clips for the Kinetics 600 dataset. The Collection is made up of about 500,000 short videos, each of which is around ten seconds long and is labeled with a single category. This dataset includes URLs for all kinds of human-related activities, such as cheering, thanking someone, etc. (Figure 24).

#### 5.4.3. HMDB-51 Dataset

**Figure 25:**
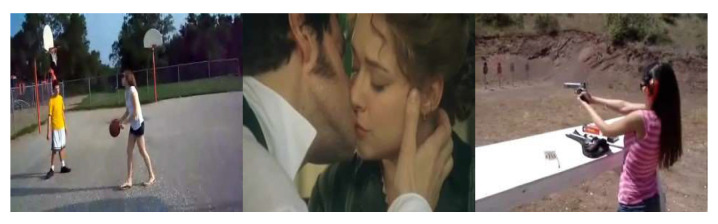
Samples from HMDB51 dataset [235].

The HMDB dataset [235], which includes approximately 7000 clips hand-labeled and manually collected from diverse sources, like YouTube, Google videos, and Prelinger collection, was released by Serre Laboratory at Brown University in 2011. Human action recognition’s 51-class dataset is broken down into five motion categories, which are defined as follows: human interaction, body movement, facial expression, object manipulation, and object interaction. Background noise and shaky camerawork are two of the most problematic aspects of using actual video footage (Figure 25).

#### 5.4.4. HOLLYWOOD Dataset

The dataset includes varied video clipsm and was first introduced by the INRIA Institute in France in 2008 [236]. Every sample is marked with one of eight activities: getting in or out of a vehicle, answering a phone call, handshaking, hugging, sitting, sitting up, standing up, and kissing. The dataset was sourced from 32 movies: 20 of the movies produced a test set, while the rest of the 12 movies provided the training sets.

#### 5.4.5. HOLLYWOOD 2 Dataset

**Figure 26:**
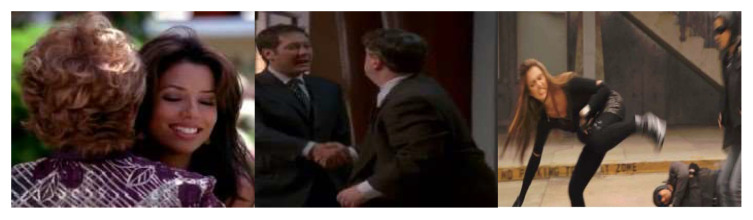
Samples from HOLLYWOOD 2 dataset [216].

This was released in 2009 by INRIA as well, to expand the Hollywood dataset [216]. It includes 12 action types (similar to the Hollywood dataset but with four additional actions: driving a vehicle, getting in a car, eating, and fighting) and a total of 3669 video clips collected over 69 movies and approximately 20 h of footage (Figure 26).

#### 5.4.6. UCF-101 Action Recognition Dataset

In 2012, the UCF CRICV (Center for Research in Computer Vision) created this [237]. The UCF101 dataset serves as an expansion to the UCF50 dataset [217], which provides 50 action classes. A total of 13,320 videos from 101 real-world action classes were gathered from YouTube and combined into one dataset. This provides the greatest range of motion and different perspectives (point of view, lighting circumstances, etc.)

Table 4 represents the details of popular datasets in the field of human action recognition.

## 6. Evaluation Metrics and Performance

Human activity recognition has adapted and utilized a number of performance indicators from other classification domains. Here, we cite commonly used measures, including precision, recall, F score, accuracy, and the confusion matrix, based on [238]. In the context of action recognition metrics, the terms “true positive”, “false positive”, “true negative”, and “false negative” have the following meanings:

- True Positive: Both the predicted and actual activity categories are the same.
- False Positive: activities that do not match the searching category but are projected to belong to the sought category.
- True Negative: activities in which the actual, as well as projected, activity do not conform to the searching class.
- False Negative: activities that should go into a certain category but are, instead, expected to fall outside of that category.

**Figure 27:**
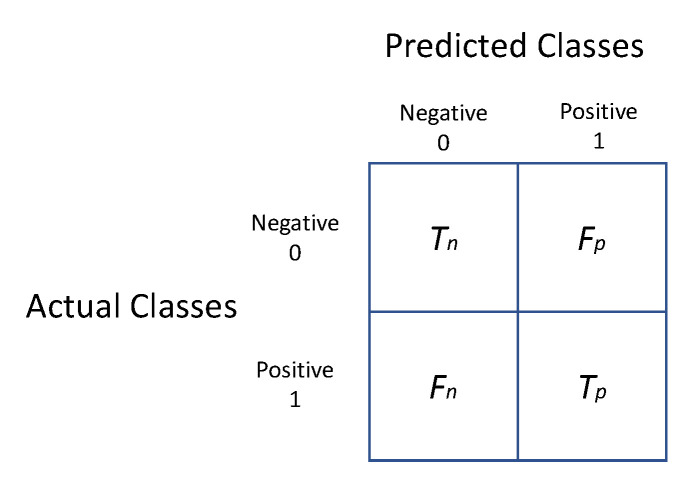
Confusion Matrix Structure.

The following is a list of the most commonly used performance metrics:

1. Recall: Recall is also known as sensitivity, true positive rate, or likelihood of detection. It is based on real positive instances, expected to be positive in advance. Sensory assesses the percentage of activities that are projected to be in a certain class. In the same way, the system’s inability to detect activities is determined by the system’s sensitivity. Mathematically, we may write this as follows:
  (1) $$Recall=T_{p}/\left(T_{p}+F_{n}\right)$$
  where *T_p_ = True positive, T_n_ = True negative, F_p_ = False positive, F_n_ = False negative.*
2. Precision: It defines the probability of an activity being observed to really occur. The likelihood that an observed activity would be wrongly identified by the recognizer is given a precision of 1. Mathematically, we may write this as follows:
  (2) $$Precision=T_{p}/\left(T_{p}+F_{p}\right)$$
3. F Score: Precision and recall are the two factors that define the harmonic mean. It tells us how accurate the test is. F measures both the accuracy and the robustness of a classifier at the same time. The value 1 is the greatest value, while 0 is the worst. Mathematically, we may write this as follows:
  (3) $$FScore=\frac{2*Recall*Precision}{Recall+Precision}$$
4. Accuracy: This metric measures the proportion of accurate predictions against the total number of samples. As long as the classes are evenly sampled, the accuracy yields satisfactory outcomes. It is possible to represent this mathematically as follows:
  (4) $$Accuracy=\frac{Number\;of\;Correct\;Predictions}{Total\;Number\;of\;Predictions}$$
  (5) $$Accuracy=\frac{T_{p}+T_{n}}{Total\;Number\;of\;Samples}$$
5. Confusion Matrix: Known as an “error matrix”, this sums together the model’s prediction outcomes and indicates the model’s overall accuracy. An error graph is generated and shown in a confusion matrix for each kind of misclassified data. There is a row for each anticipated class and a column for each actual class in the matrix, or the other way around. Figure 27 shows the structure of a confusion matrix.

The methods that achieved the highest accuracies on popular datasets are shown in Table 5.

## 7. Research Issues, Opportunities, and Future Directions

### 7.1. Research Issues

In this section, several problems that may impair the functioning of HAR systems are discussed. At different stages of the recognition process, several techniques may be employed. The systems in question are mostly linked to equipment for acquiring and processing data, as well as experimental and application spaces. An image-based recognition system’s primary challenge is lighting fluctuation, which has an impact on the quality of pictures and, thus, on the information that is processed. Systems designed to function utilizing a single viewpoint acquisition device impose a similar restriction on how the perspective may be altered. The more information that can be collected, the more restricted and granular the visualization is for the actions being studied. The following encompasses a number of the various kinds of occlusion: self-occlusion, when body parts obscure each other, other-object occlusion and partial-body-part occlusion. These are key constraints to wearable augmented reality systems. The efforts of [10,84,221,222,248,249] addressed these issues. The diversity of gestures associated with complex human actions, and the existence of links between comparable kinds of actions, may introduce complications owing to data association issues. In order to create comprehensive, resilient, and adaptable HAR systems, it is essential to identify and correct any shortcomings. For example, [10,220,222,224,249], which described the limitations in hand configurations, preset activities, and detection of basic motions and actions. Certain techniques for the identification of body parts and items in scenes may mistake the person’s body parts with materials in the scene, as shown in [222,224,249,250], or malfunction when people wear different clothes [10,84]. These issues are linked to other issues, including the amount of background noise [222], complicated or shifting backdrops, and unstructured scenery [10,84,221,248,249,250], as well as changes in size [10,84]. Many researchers evaluate the performance of their ideas using their own recorded datasets. Benchmark datasets are limited to domain-specific applications, which presents issues. For instance, the everyday activities and fall recognition datasets utilized for training successful models are just too small.

### 7.2. Opportunities

A contemporary system of human action recognition (HAR) has many complications that have to be managed in order to fulfil the primary tasks for which they were designed. A HAR-based video surveillance system may be installed as long as it is continuously monitored and generates stable responses that appear on time. In [222,224,249] this problem was addressed. There is an even bigger problem, when trying to represent human-to-human and human-to-object interactions with precision, that is not as simple as it seems. This may be used in security and surveillance systems, and may be able to spot many odd situations.

At the same time, there are new social problems that have emerged as a result of increased implementation costs and adoption for surveillance, elderly support, and patient monitoring, combined with society’s greater acceptance. One specific example, in [220,222], illustrates how difficult it is to integrate devices at home for monitoring, which is regarded as an invasion of privacy and intimacy. It is also important to investigate the HAR system’s progress in mobile devices. In order to satisfy the user’s privacy constraints, this method of recording data would require storing the information on the user’s device, faster server-to-device communication, and shortened computing time. Battery life is a big problem, and on-device implementation is tough owing to memory constraints, identification model parameter space constraints, and power consumption [251]. The third difficulty is associated with the limits of the user’s physiological and physical capabilities, since the user depends on these systems for movement and functioning. According to this logic, the usage of HAR technologies should not be dependent on the user’s age, race, body size, or capability. It should be possible for both novice and expert user to get the most out of these tools. The problem of achieving large gains is acknowledged in [222,249]. The detection of continuous motion is made much more challenging due to the amount of data that is being streamed at any one time. With all due respect, it is no wonder that HAR systems have not yet been able to recognize and identify diverse motions in varied lighting situations and are not willing to develop with respect to the rate and quantity of gestures. The main issue addressed in numerous academic publications includes such titles as [10,84,222,224,249]. HAR systems are capable of context awareness. Therefore, further investigation is needed in this field. This may be beneficial in promoting the usage of previously suggested methods and the progress that has been achieved in various application areas.

Long-term videography may be particularly difficult to understand and identify when it comes to day-to-day activities. This is because everyday life activities are made up of many complicated actions, which is why long-term films include them. Even though these actions are diverse and varied, they are challenging to model. A further problem exists in that beginning and finishing times overlap for each activity. This issue is discussed by [248]. In addition, resolving the difficulty of discriminating between acts that are voluntary and involuntary is still an active topic to explore.

In addition to the above difficulties, additional general challenges related to human activities, such as missing portions of a video, recognizing multiple activities being done by the same person, and identifying and predicting actions, such as in congested settings, are addressed in [219,252]. The key underlying problems in deep learning-based HAR technologies include the presence of memory constraints, an abundance of parameters to be updated, complex multi-variant data collection and fusion, and the implementation of multiple deep learning-based architectures in smartphones and wearable devices.

### 7.3. Future Directions

Identifying human actions with the use of multi-modal data has several benefits, primarily because it often offers more detailed information. It can also be utilized to reduce the amount of noise present in data from single sources; thus, increasing the reliability of action identification. As a result, in order to improve the effectiveness of future studies of human actions, farther efficient incorporation of varied intelligence should be created, rather than the repetitive unification of characteristics from various origins. It is possible that, in the case of human interactions, the combination of characteristics of people, and correlations derived from different data sources, would result in a more reliable interpretation. Aside from that, relational content from the surrounding environment, which has been relatively understudied, has the capability to boost the efficiency of conventional feature representations for human action identification.

The ability to adjust our view for various camera positions is a beneficial feature, since it mostly enables us to move about and simplifies the process of calibrating sensors located in different places. The skeleton-based techniques are inherently resistant to being seen from various perspectives. Nevertheless, the computed skeleton data may not be correct when viewed from the side, which most likely results in a decrease in recognition performance. In this approach, the recent approaches to depth-based techniques are mostly focused on the generation of artificial multi-view data in order to supplement the training set. As a result, prospective studies might put greater emphasis on the development of feature descriptors that are perspective invariant.

The current understanding of activity recognition algorithms is incomplete, because depth-based datasets are often produced through certain contexts. One may find a significant discrepancy between the compiled datasets and the real world, because of the absence of categories, samples, occlusion occurrences, restrained activities, distance, and internal environmental variables. Due to this, algorithms are challenging to use in real-world circumstances. For this reason, it is important to gather large-scale datasets for training and testing to use in real situations.

Although many powerful deep learning techniques may beat hand-crafted techniques, the majority of them require the use of a pre-processing phase that involves color, depth, or skeleton data, before extracting hand-crafted representations. While handmade representations are primarily dedicated to representing feature dimensions, they also reduce the capacity of deep learning techniques to understand the results. It could be because of a lack of samples for training. Thus, if we provide enough training data, future models may come up with new types of deep learning architectures with the express purpose of directly learning representations from raw video data.

Human behavior analysis from a live video stream is sought after in practical applications, where the activity identification algorithms, which are applied to pre-segmented video sequences, must be very precise. This means that, since existing research studies tend to use trim data with a single category per segment, we do not yet know whether, or if, their findings are applicable to online instances. One important path to follow while creating identification methods is to identify recognition techniques that may be used in real situations.

The term “human behavior”, which refers to the various factors involved in human activity, such as facial expressions, human behavior, and attention, is more complex than the terms “human action” or “human interaction”. Automated behavior interpretation is an important component in the development of genuine intelligent systems, and it benefits a certain field that looks at human cognitive capabilities.

## 8. Conclusions

Many areas of computer vision, like human–computer interaction, robotics, monitoring, and security, require understanding and interpreting human actions efficiently. This article provides an overall look at the current advances in this research area. It sets forth many criteria, according to which it classifies items. This paper began by discussing the various HAR systems and the systems’ primary goals. Following this, it provided a summary of the procedures that are currently considered “state-of-the-art”, including the validation procedures that utilized to test those approaches. In addition to this, it classified human actions, as well as the methodologies used to represent particular action information. The various techniques might also be classified according to the kind of acquisition equipment, and an additional category based on the stages of recognition is provided (detection, tracking, and recognition). When looking at the results of this research, it was found that every methodology suffers from some constraints. This may be attributed to progress in the deep learning method and positive findings with respect to detection and identification performance. Alternatively, group activities and interactions are important study subjects, because they may offer relevant information in a wide variety of HAR domains, such as public security, camera monitoring, and the identification of aberrant behavior. An extension of HAR processes to smartphones is also being investigated, since cellphones have become more essential to our everyday lives and are already accepted by society, despite their lack of invasive properties. In order to create a good human action recognition system, we need to consider a number of characteristics, encode them into a distinct model, and ascertain that the outcomes of the modeling are correct. Finally, as a closing statement, we may state that, although many new methods with the goal of better understanding human activity have been created, they still face many difficulties that must be overcome.

## Figures and Tables

**Table 1:** Handcrafted feature-based state-of-the-art methods for action recognition.

Methods	Data Type	Dataset	Performance	Source	Year
Fast Fourier Transform	RGB	UCF101 Kinetics	Acc: 99.21 Acc: 98.24	[94]	2022
QSVM	RGB	UCF11 HMDB51	Acc: 94.43 Acc: 87.61	[95]	2021
SVM	RGB	UCSDped-1 UCSDped-2 UMN	Acc: 97.14 Acc: 91.13 Acc: 95.24	[96]	2017
SVM	RGB	UCF11 UCF50	Acc: 78.6 Acc: 72.9	[97]	2014
SVM	RGB	MSRAction3D UTKinectAction CAD-60 MSRDailyActivity3D	Acc: 94.3 Acc: 91.9 Acc: 87.5 Acc: 80.0	[98]	2014
SVM	RGB	Weizmann KTH Hollywood2	Acc: 100 Acc: 96.3 Mean Average Precision: 58.46	[99]	2011
SVM	RGB	KTH Weizmann i3Dpost Ballet IXMAS	Average Acc.: 95.5 Average Acc.: 100 Average Acc.: 92.92 Average Acc.: 93.25 Average Acc.: 85.5	[100]	2016
SVM	RGB	KTH UCFSports Hollywood2	Average Acc: 91.8 Average Acc: 94 Mean Average Precision: 68.1	[101]	2018
SVM with ASAGA	RGB	UCSDped 1	Acc: 87.2	[102]	2014
SVM with PSO	Skeleton	MSRAction3D UTKinect Florence3D action	Acc: 93.75 Acc: 97.45 Acc: 91.20	[103]	2016
SVM with GA	RGB	KTH HMDB51 UCF youtube Hollywood2	Acc: 95.0 Acc: 48.4 Acc: 82.3 Acc: 46.8	[104]	2015
SVM-Neural Network	RGB	KTH Weizmann	Average Acc.: 96.4 Average Acc.: 100	[105]	2015
RF	Skeleton	UTKinect	Acc: 92	[106]	2013
NBNN	3D joints skeleton	MSRAction3D-Test1 MSRAction3D-Test2 MSRAction3D-cross-subject	Acc: 95.8 Acc: 97.8 Acc: 83.3	[21]	2014
HMM-Kernel Discriminant Analysis	Silhouette	Elder care data	Acc: 95.8	[107]	2011
HMM	Skeleton	Im-DailyDepthActivity MSRAction3D (CS) MSRDailyActivity3D (CS)	Acc: 74.23 Acc: 93.3 Acc: 94.1	[49]	2017
Dynamic Time Wrapping	RGB	MuHAVi (LOSO) MuHAVi (LOAO)	Acc: 100 Acc: 100	[108]	2014
KELM	Depth	MSRGesture (LOSO) MSRAction3D (CS)	Acc: 93.4 Acc: 91.94	[109]	2015
KELM	Depth	DHA MSRAction3D MSRGesture3D MSRDailyActivity3D	Acc: 96.7 Acc: 96.70 Acc: 99.39 Acc: 89	[65]	2017

**Table 2:** CNN feature based state-of-the-art methods for HAR.

Methods	Data Type	Dataset	Performance	Source	Year
PoseConv3D	RGB+Depth	NTU-RGBD	Acc: 97.1	[80]	2022
Temporal Difference Networks	RGB	Something-SomethingV1 Kinetics	Acc: 68.2 Acc: 79.4	[123]	2021
CNN	RGB	UCF101 HMDB51 FCVID ActivityNet	Acc: 98.6 Acc: 84.3 Acc: 82.1 Acc: 84.4	[124]	2020
2-stream Convolution Network	RGB	UCF101 HMDB51	Acc: 91.5 Acc: 65.9	[27]	2015
3-stream CNN	RGB	KTH UCF101 HMDB51	Acc: 96.8 Acc: 92.2 Acc: 65.2	[125]	2017
Multi-stream CNN	Skeleton	NTU-RGBD (CS) NTU-RGBD (CV) MSRC-12 (CS) Northwestern-UCLA	Acc: 80.03 Acc: 87.21 Acc: 96.62 Acc: 92.61	[126]	2017
3D CNN	RGB	KTH	Acc: 90.2	[127]	2012
Actional-graph-based CNN	Skeleton	NTU-RGBD (CS) NTU-RGBD (CV) Kinetics Kinetics	Acc: 86.8 Acc: 94.2 Top-5 acc: 56.5 Top-1 acc: 34.8	[128]	2019
CNN	RGB	UCF101 HMDB51	Acc: 92.5 Acc: 65.2	[129]	2016
CNN	RGB	UCF50 UCF101 YouTube action HMDB51	Acc: 96.4 Acc: 94.33 Acc: 96.21 Acc: 70.33	[130]	2019
CNN-Genetic Algorithm	RGB	UCF50	Acc: 99.98	[131]	2016
CNN	Skeleton	UTD-MHAD NTU-RGBD (CV) NTU-RGBD (CS)	Acc: 88.10 Acc: 82.3 Acc: 76.2	[110]	2017
ConvNets	RGB	CIFAR100 Caltech101 CIFAR10	Acc: 75.87 Acc: 95.54 Acc: 91.83	[132]	2017
Temporal CNN	Skeleton	NTU-RGBD (CV) NTU-RGBD (CS)	Acc: 83.1 Acc: 74.3	[133]	2017
ConvNets	Skeleton	MSRAction3D UTKinect-3D SBU-Kinect Interaction	Acc: 97.9 Acc: 98.5 Acc: 96.2	[134]	2019
DBN and CNN	Skeleton	HMDB51 Hollywood 2	Acc: 80.48 Acc: 91.21	[135]	2017
CNN-LSTM	Skeleton	NTU-RGBD (CV) NTU-RGBD (CS)	Acc: 90.10 Acc: 82.89	[136]	2017
3D-ConvNets-LSTM	Depth	NTU-RGBD(CV) NTU-RGBD(CS) UCLA	Acc: 95.4 Acc: 93 Acc: 93.1	[137]	2019

**Table 3:** Hybrid feature based state-of-the-art methods for HAR.

Method	Data Type	Dataset	Performance	Source	Year
HyRSM	RGB	UCF101	Acc: 93.0	[175]	2022
GCN	Skeleton	NTU-RGBD	Acc: 96.1	[176]	2022
PYSKL	Skeleton	NTU-RGBD UCF101	Acc: 97.4 Acc: 86.9	[177]	2022
ActionCLIP	RGB+Text	Kinetics	Acc: 83.8	[178]	2021
IMGAUD2VID	RGB+Audio	ActivityNet	Acc: 80.3	[179]	2020
AGCN-LSTM	Skeleton	NTU-RGBD(CS) NTU-RGBD(CV) UCLA	Acc: 89.2 Acc: 95 Acc: 93.3	[180]	2019
Stacked LSTM	Skeleton	SBU Kinect HDM05 CMU	Acc: 90.41 Acc: 97.25 Acc: 81.04	[144]	2016
Stacked LSTM	Skeleton	MSRDailyActivity3D NTU-RGBD (CS) CAD-60	Acc: 91.56 Acc: 64.9 Acc: 67.64	[181]	2018
Stacked LSTM	RGB	HMDB51 UCF101 Hollywood2	Acc: 41.31 Acc: 84.96 MAP: 43.91	[182]	2015
Differential RNN	RGB and Skeleton	MSRAction3D (CV) KTH-1 (CV) KTH-2 (CV)	Acc: 92.03 Acc: 93.96 Acc: 92.12	[138]	2015
TSN	RGB	HMDB51 UCF101	Acc: 69.4 Acc: 94.2	[183]	2016
FCN	RGB	Sports Video	Acc: 97.4	[184]	2019
AGCN	Skeleton	NTU-RGBD (CS) NTU-RGBD (CV) Kinetics Kinetics	Acc: 88.5 Acc: 95.1 Top 5% acc: 58.7 Top 1% acc: 36.1	[44]	2019
Two-stream MiCT	RGB	HMDB51 UCF101	Acc: 70.5 Acc: 94.7	[185]	2018
DBN	Depth	MHAD MIVIA	Acc: 85.8 Acc: 84.7	[186]	2014
GAN	RGB	UCF101 HMDB51	Acc: 47.2 Acc: 14.40	[187]	2018

**Table 4:** Polular Public Dataset.

Dataset	Input Type	Action Type	#Classes	#Videos	Year	Ref.
HMDB51	RGB ^1^	Group	51	6766	2011	[235]
UCF101	RGB	Group	101	13,320	2012	[237]
NTU RGB + D	RGB + D ^2^ + S ^3^	Atomic	60	56,880	2016	[142]
ActivityNet	RGB	Group	200	19,994	2016	[233]
Kinetics	RGB	Group	400	306,245	2017	[234]
Hollywood2	RGB	Group	12	1707	2009	[216]
KTH	RGB	Atomic	6	2391	2004	[230]
UCF50	RGB	Interaction	50	6618	2012	[217]
MSR Daily Activity 3D	RGB + D + S	Interaction	16	320	2012	[87]
MSR Action 3D	D + S	Atomic	20	567	2010	[63]
MuHAVI	RGB	Interaction	17	1904	2010	[232]
MCAD	RGB	Behavior	18	14,298	2016	[218]

^1^ Red, Green, Blue. ^2^ Depth. ^3^ Skeleton.

**Table 5:** Accuracies on Polular Dataset.

Action Types		Accuracies	Method	Year
**Atomic Action**				
	KTH	99.86%	PredRNN-V2 [239]	2021
	NTU RGB+D	97.1%	PoseC3D [80]	2022
	MSR Action 3D	98.02%	Temporal Subspace Clustering [240]	2021
**Behavior**				
	MCAD	86.9%	Conflux LSTMs network [241]	2021
**Interaction**				
	MSR Daily Activity 3D	97.5%	DSSCA-SSLM [242]	2017
	MuHAVI	100%	ST-LSTM (Tree) + Trust Gate [26]	2016
	UCF50	94.4%	MIFS [243]	2015
**Group Activities**				
	ActivityNet	96.9%	Text4Vis (w/ViT-L) [244]	2023
	Kinetics	91.1%	InternVideo-T [245]	2022
	HMDB-51	87.56%	DEEP-HAL with ODF+SDF [246]	2021
	Hollywood2	71.3%	DS-GRU [247]	2021
	UCF-101	98.64%	SMART [124]	2020

## Data Availability

Not applicable.
